# Automated caries detection in vivo using a 3D intraoral scanner

**DOI:** 10.1038/s41598-021-00259-w

**Published:** 2021-10-28

**Authors:** Stavroula Michou, Mathias S. Lambach, Panagiotis Ntovas, Ana R. Benetti, Azam Bakhshandeh, Christos Rahiotis, Kim R. Ekstrand, Christoph Vannahme

**Affiliations:** 1 Department of Odontology, University of Copenhagen, 2200 Copenhagen, Denmark; 2 3Shape TRIOS A/S, 1060 Copenhagen, Denmark; 3 School of Dentistry, National and Kapodistrian University of Athens, 11527 Athens, Greece

**Keywords:** Dental caries, Imaging and sensing

## Abstract

The use of 3D intraoral scanners (IOS) and software that can support automated detection and objective monitoring of oral diseases such as caries, tooth wear or periodontal diseases, is increasingly receiving attention from researchers and industry. This study clinically validates an automated caries scoring system for occlusal caries detection and classification, previously defined for an IOS system featuring fluorescence (TRIOS 4, 3Shape TRIOS A/S, Denmark). Four algorithms (*ALG1, ALG2, ALG3, ALG4*) are assessed for the IOS; the first three are based only on fluorescence information, while *ALG4* also takes into account the tooth color information. The diagnostic performance of these automated algorithms is compared with the diagnostic performance of the clinical visual examination, while histological assessment is used as reference. Additionally, possible differences between in vitro and in vivo diagnostic performance of the IOS system are investigated. The algorithms show comparable in vivo diagnostic performance to the visual examination with no significant difference in the area under the ROC curves ($p>0.05$). Only minor differences between their in vitro and in vivo diagnostic performance are noted but no significant differences in the area under the ROC curves, ($p>0.05$). This novel IOS system exhibits encouraging performance for clinical application on occlusal caries detection and classification. Different approaches can be investigated for possible optimization of the system.

## Introduction

The use of 3D intraoral scanners (IOS) and corresponding software for oral disease detection and monitoring has proven potential^1–5^. There is increasing development in this area, both by companies that produce medical devices, and by researchers seeking improved devices and software that can support automated detection and objective monitoring of oral diseases such as caries, tooth wear and periodontal diseases either in a clinical setup or remotely^1–12^.

The implementation and diagnostic performance of an automated caries scoring system based on the fluorescence method using blue-violet light (415 nm wavelength) in a 3D IOS system (TRIOS 3, 3Shape TRIOS A/S, Denmark) has previously been investigated^1^. Different caries classification algorithms were investigated for this fluorescence-based IOS system, showing good in vitro diagnostic performance when assessing occlusal caries lesions. More specifically, at the caries stages where the comparison between the conventional methods and the IOS caries classification system was possible, the best-performing IOS algorithms employing optimal cut-offs showed slightly higher sum of Sensitivity (SE) and Specificity (SP) (SE+SP = 1.58–1.84) than the visual-tactile examination (SE+SP = 1.73–1.81) and significantly higher than the radiographic examination (SE+SP = 1.37–1.78). The only exception was observed for the caries lesions located in the outer third of dentin, where the radiographic assessment showed a higher value (SE+SP = 1.78) compared to the best-performing IOS algorithms (SE+SP = 1.67–1.69). In that study, both visual-tactile and radiographic assessments employed the International Caries Detection and Classification System criteria (ICDAS)^1,13^. The idea behind the development of the automated caries detection and classification system to accompany the 3D IOS is that by combining a method similar to the well-documented Quantitative Light-Induced Fluorescence (QLF)^6,8,14,15^ with the 3D information provided by the IOS, detection and monitoring of the caries lesions can potentially be improved.

The study mentioned above^1^ and later investigations led to the definition of different algorithms for an automated caries scoring system, and its implementation in a prototype software accompanying the 3D IOS (TRIOS 4, 3Shape TRIOS A/S, Denmark). These algorithms employ red and green fluorescence ($R_{fluo}$, $G_{fluo}$) signal^1^ resulting from scanning with light at 415 nm. However, tooth color (Red, Green and Blue) signal (*R, G, B*) is simultaneously obtained from scanning the teeth with white light. It is speculated that by combining all the available color information on a 3D model, and by analyzing any difference of color signal intensity on the tooth surface together with the fluorescence changes corresponding to sound and demineralized dental tissue, the accuracy in detecting occlusal caries lesions could be increased^12,16,17^. This hypothesis is supported by previous research, in which similar approaches combining the fluorescence method with reflectance enhancement showed relatively accurate detection and monitoring of caries lesions in vitro (SE+SP = 1.55)^16,17^. Thus, an algorithm combining all the color information on the 3D model ($R_{fluo}$, $G_{fluo}$, *R, G, B*) was defined and tested on existing sample^1^. This specific algorithm showed the best in vitro diagnostic performance for occlusal caries detection and classification at one optimal cut-off in enamel and two in dentin (area under the ROC curve, $A_z$ $>0.9$, SE $>0.83$ and SP $>0.87$), which motivated us to include it in this validation study.

Based on the diagnostic performance of the 3D IOS for in vitro occlusal caries detection^1^ and considering the unique advantage of 3D models, which combine geometry, color signal from the tissues, and, in this case, fluorescence signal, we assume that this device can help to overcome some limitations observed for the existing 2D intraoral cameras featuring fluorescence for caries detection. For example, difficulties in obtaining reproducible 2D intraoral images for monitoring caries lesions over time is a common issue, limited largely by the image acquisition angle. The latter can significantly affect the size of the lesion depicted on the 2D images^18^ but is expected to have less influence on the assessment using 3D models where the averaging of image data gives less noise and eliminates images obtained from steep angles.

Despite the good results obtained for the IOS system in vitro^1^, it was essential to validate the defined algorithms and corresponding cut-offs on a new blind sample in vivo^19^. Previous studies assessing other devices featuring fluorescence for caries detection have observed significant differences among the devices’ in vitro diagnostic performance at optimal cut-offs and their subsequent performance achieved in in vivo validation studies, where pre-defined cut-offs were assessed on independent samples^19–22^. The latter has led previous researchers to the conclusion that the in vitro defined cut-offs need modification for in vivo application.

### Aim

The purpose of this study was to clinically validate four automated caries scoring system algorithms previously defined for the IOS system, using histological assessment as reference method. Further aims were: (i) to compare the performance of the automated scoring system with the clinical examination employing the ICDAS criteria; and (ii) to assess possible differences in the performance of the automated system under in vitro and in vivo conditions.

## Materials and methods

### Study sample

Sample size calculation was done using the formula described by Burderer^23^, for a confidence interval at 95%, absolute error at 0.1, and based on the expected diagnostic performance for the IOS system (SE $\ge$ 0.84, SP $\ge$ 0.76)^1^. These values were based on the performance of the investigated device in the literature^1^. This calculation resulted in a minimum of 100 examination sites that should be included in the current study.

Permanent molars and premolars scheduled for extraction at the surgery department of the School of Dentistry of the University of Copenhagen were considered for inclusion in the study. The age range of patients was from 18 to 60 years old. Teeth with severe developmental defects, calculus on the occlusal surface, visible extensive caries lesions on other surfaces than the occlusal, and restored teeth were not included in the sample. According to these criteria, 58 teeth scheduled for extraction were selected for examination.

### Ethics

This clinical study received ethical approval from the Research Ethics Committee of the School of Dentistry of the National and Kapodistrian University of Athens, Greece (prot. nr. 423/08.07.2019). The study was conducted in accordance with the declaration of Helsinki and the General Data Protection Regulation (GDPR). All clinical steps and scanning of the extracted teeth were conducted at the aforementioned University. Thereafter, all extracted teeth were fully anonymized and sent for histological analysis to the University of Copenhagen, Denmark. According to the rules in Denmark, research projects involving completely anonymous or anonymized human biological material, which is collected in accordance with the legislation at the collection site, are exempted from notification to the Danish Committee system (cf. Article 14(3) of the Committees Act).

All study participants gave informed consent and agreed to publish anonymized information or images in an online publication.

### Study design

**Figure 1:**
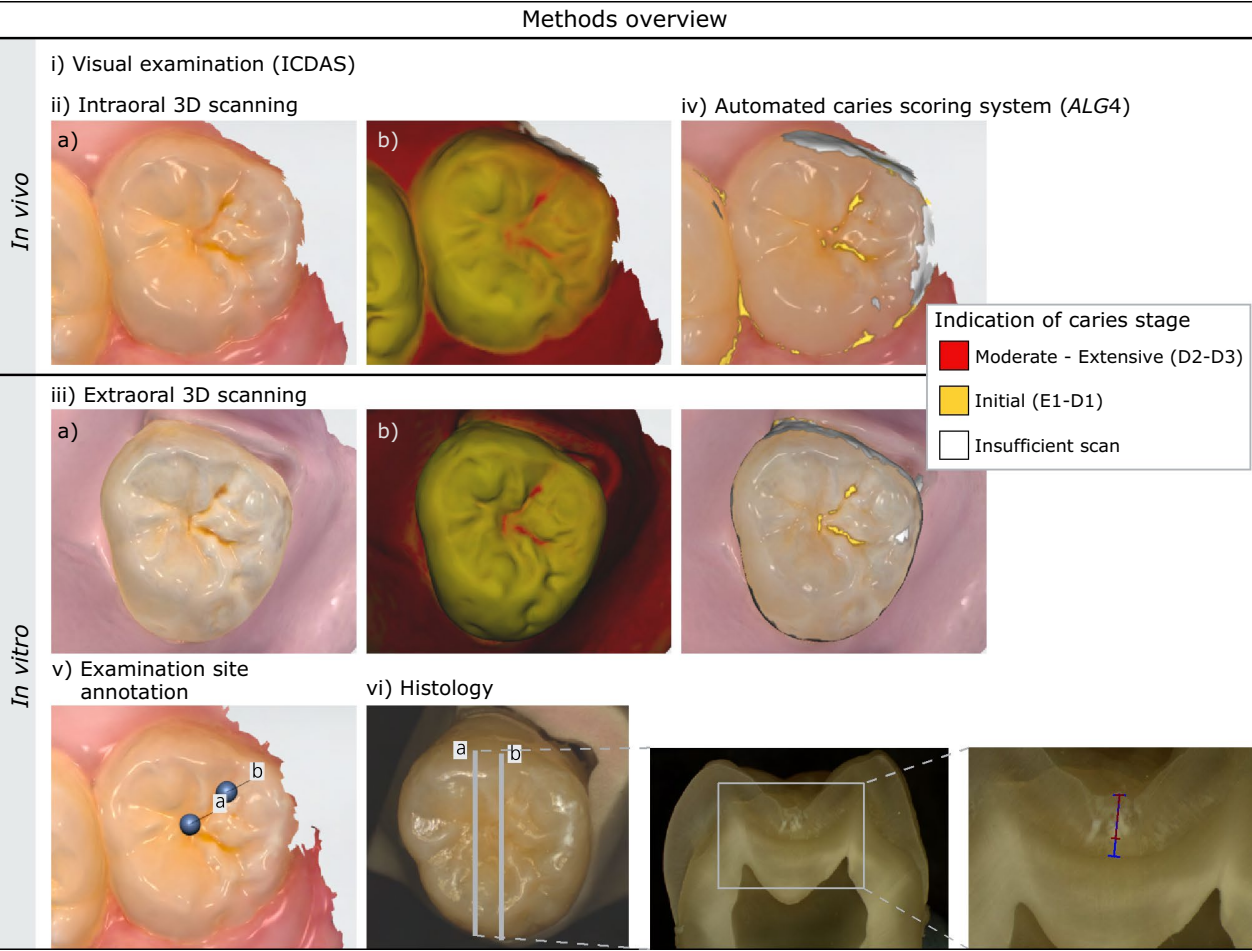
Study methods overview in vivo and in vitro. 3D models of the same tooth scanned (iia) in vivo and (iiia) in vitro using white light; the tooth color signal was mapped onto the models. The same tooth was scanned (iib) in vivo and (iiib) in vitro with the 415 nm wavelength light, which excites fluorescence from the dental tissues. (iv) caries score indication based on TRIOS patient monitoring software (3Shape TRIOS A/S, Denmark) according to *ALG4*. Indication of caries stages: Initial; caries lesions in enamel and outer third of dentin (Histology E1–D1). Moderate–extensive; caries lesions in middle and inner thirds of dentin (Histology D2–D3). Insufficient scan: insufficient data on tooth color and/or fluorescence that does not allow the automated caries score calculation. (v) selected examination sites (**a**,**b**) annotated on the 3D model. (vi) tooth sectioning lines corresponding to the examination sites (**a**,**b**) for the histological assessment. On the tooth section, the red measurement line corresponds to the demineralization depth and the blue measurement line corresponds to the enamel thickness.

The overall study workflow is presented in Fig. 1.

This in vivo study with in vitro validation assessed four different algorithms (*ALG1*–*ALG4*) implemented in the IOS system for automated caries detection and classification. 3D models of the examined teeth were obtained both in vivo and in vitro, i.e. before and after tooth extraction, in order to assess any possible differences in the algorithms’ performance in different conditions. The latter could potentially help draw some conclusions regarding the validity of the in vitro caries detection results obtained for this 3D IOS system, and the in vivo applicability of in vitro results. Additionally, a visual-tactile examination using the ICDAS criteria^24^ was conducted in vivo and histological assessment was used as reference test in vitro (Table 1). Information regarding the examiners’ calibration and blinding are provided in the supplementary material.

### Visual examination (ICDAS)

The clinical examiner (P.N.) defined one to three examination sites in the occlusal pits and fissures of each selected tooth and examined all teeth in vivo employing the visual ICDAS criteria for caries classification^13,25,26^. Examination was performed on dry surfaces, under proper illumination and after polishing of the occlusal surfaces with prophylactic brushes and a low-speed handpiece (Kavo Intra 20k). One score (ICDAS0–ICDAS6) was assigned to each examination site, and after the 3D model acquisition, the exact position of the examination site was annotated on the 3D model Fig. 1v.

### 3D scanning

Subsequent to visual examination, all teeth were scanned in vivo using the 3D IOS TRIOS 4 (3Shape TRIOS A/S, Denmark) aided by commercial software (TRIOS vers. 1.18.2.11 and Dental Desktop vers. 1.6.8.1, 3Shape TRIOS A/S, Denmark) and according to the manufacturer’s recommendations: the dental lamp was switched off, other external light was limited as much as possible^27^, teeth surfaces were clean and dry and the recommended scanning strategy was followed.

First, by scanning with white light, a digital 3D model of the teeth with tooth color texture was created (Fig. 1iia). Then, by scanning a second time using light at 415 nm, a texture representing the fluorescence signal received from the tissues was mapped onto the 3D model (Fig. 1iib). The intraoral scanning procedure was finalized when sufficient tooth color and fluorescence information was obtained according to the software’s indication.

Following in vivo intraoral scanning, the teeth were extracted and transferred shortly thereafter to the laboratory for in vitro scanning. There, the teeth were mounted on individual bases made of putty impression material (Zetalabor, Zhermack, Italy) and scanned again with the same IOS system, following the same procedures described for the intraoral scanning in vivo. The in vitro models were obtained in a dark room (Fig. 1iiia,b), within 48 hours from tooth extraction.

### Intraoral scanner’s algorithms

Four different algorithms (*ALG1*–*ALG4*) defined for caries detection and classification on the 3D models were assessed. An article describing the definition of the first three algorithms (*ALG1*–*ALG3*) was published previously by Michou et al.^1^. Mathematical functions $f_2$–$f_4$ of the mentioned study correspond to *ALG1*–*ALG3* in the current study. The last algorithm, *ALG4*, was defined at a later stage using the same sample and methods as described in the above mentioned study^1^. Histology was used as the reference method for the definition of all algorithms. Receiver Operating Characteristic (ROC) analyses were conducted on the raw data from each algorithm. Optimal cut-offs for different caries severity levels according to histology were defined by the sum of SE and SP at each histological level (Table 1).

For *ALG1* and *ALG2*, reliable independent cut-offs (SE+SP $>1.7$) could only be defined for two caries severity levels: (i) caries lesions in enamel ($\ge$ E1) and (ii) caries lesions in dentin ($\ge$ D1). For *ALG3* and *ALG4*, an additional cut-off corresponding to (iii) caries lesions in the middle-inner third of dentin ($\ge$ D2) was also defined. Thus, using *ALG3* and *ALG4* the lesions in the outer third of dentin received a different score than the lesions in the middle-inner third (Table 1). The first three algorithms (*ALG1–ALG3*) were based exclusively on the fluorescence signal received by the dental tissues. More specifically: *ALG1* represents the absolute green fluorescence signal ($G_{fluo}$) on each examination site; *ALG2* represents again the $G_{fluo}$ but taking as reference the average $G_{fluo}$ from the sound surfaces on the same tooth; and *ALG3* represents both red ($R_{fluo}$) and green fluorescence signal ($G_{fluo}$) on the examination sites and uses as reference the average $R_{fluo}$ and $G_{fluo}$ from sound surfaces located on the same tooth. The last algorithm, *ALG4*, was found by logistic regression, and takes into account both fluorescence ($R_{fluo}$, $G_{fluo}$) and tooth color signal (R, G, B) from the examination sites using the sound tooth surfaces as reference.

Rather than selecting the areas of interest manually in order to calculate the caries scores^1^, in the current study the prototype software already integrated the algorithms *ALG1–ALG4*. This software was based on the commercially-available TRIOS Patient Monitoring software (3Shape TRIOS A/S, Denmark) and enabled the automated display of a color overlay on the 3D models of the teeth, which represented the caries severity indication on the model according to each algorithm (Fig. 1iv).

Using this custom-made software, an independent examiner not involved in the clinical examination (S.M.) assessed the 3D models acquired both in vivo and in vitro. The automated scores given from each algorithm on the 3D models were registered on the same examination sites initially selected by the clinical examiner (P.N.). The scoring system corresponding to each algorithm is shown in Table 1.

### Reference test—Histology

Histological assessment was used as the reference standard such as that described in the literature^1^. The maximum caries lesion depth, as well as the enamel or dentin thickness (at the same position), were registered for each examination site (Fig. 1vi). Based on the outcome resulting from the fraction caries lesion depth/enamel thickness or caries lesion depth/dentin thickness for lesions located in enamel and dentin, respectively, the following histological scores were given to each examination site:

- E0 sound;
- E1 lesions in the outer half of enamel (fractions $<0.5$);
- E2 lesions in the inner half of enamel including the dentin-enamel junction (DEJ) (fractions $\ge 0.5$);
- D1 lesions in the outer third of dentin (fractions $<0.33$),
- D2 lesions in the middle third of dentin (fractions $\ge$ 0.33 and $<0.66$); and
- D3 lesions in the inner third of dentin, with or without pulp involvement (fractions $\ge 0.66$).

**Table 1:** Scoring systems employed by the different methods according to histology.

	Histology	*ALG1,2*	*ALG3,4*	Visual (ICDAS)
**SOUND**	**E0:** Sound	**0:** Sound	**0:** Sound	**0:** Sound tooth surfaces show no visible evidence of caries when viewed after cleaning and after 5 seconds of air-drying.
**ENAMEL**	**E1:** Caries in the outer half of enamel	**1:** Caries in enamel	**1:** Caries in enamel	**1:** First visual change in enamel (opacity or discoloration) visible at the entrance of pit or fissure, seen after 5 seconds of air-drying.
	**E2:** Caries in the inner half of enamel—including the dentin–enamel junction (DEJ)			**2:** Distinct visual change in enamel (opacity or discoloration) visible when both wet and dry, with no evidence of surface breakdown or underlying dentin shadowing.
**DENTIN**	**D1:** Caries in the outer third of dentin	**2:** Caries in dentin	**2:** Caries in the outer third of dentin	
	**D2:** Caries in the middle third of dentin		**3:** Caries in the middle or inner third of dentin	**3:** A white or brown spot lesion with localized enamel breakdown, without visible dentin exposure.
				**4:** Non-cavitated surface with an underlying dentin shadow, which obviously originated on the surface being evaluated
	**D3:** Caries in the inner third of dentin			**5:** Visually distinct cavity in opaque or discoloured enamel and exposed dentin.
				**6:** Extensive (more than half of the surface) and visually distinct cavity with exposed dentin.

### Data analysis

All examination sites were assigned an independent score using the different software algorithms, visual assessment (ICDAS), and histology.

Spearman’s rank correlation coefficient $(r_s)$ was used to assess possible correlation between the histology and the scores originated from algorithms or visual assessment. The diagnostic performance for all methods was expressed by ROC analyses and contingency tables using histology as reference (see Supplementary table S1). Area under the ROC curve (Az), Sensitivity (SE), Specificity (SP) and accuracy (ACC) were then calculated after dichotomizing the data at the E1, D1 and D2 histological levels, which correspond to the three cut-offs defined for the algorithms. Areas under the ROC curves for the investigated methods at the E1, D1 and D2 levels were compared pairwise using DeLong’s algorithm^28^, while SE and SP values were compared using McNemar’s test^29^. The standard error (Std. Err.) for SE and SP was adjusted for possible clustering effect as multiple examination sites were selected on the same tooth^30–32^. The McNemar–Bowker test was employed to assess possible differences between the in vivo and in vitro results for the different algorithms.

Spearman’s rank correlation coefficient, contingency tables ROC analyses, and McNemar’s test were performed using IBM SPSS Statistics (Version 26, IBM Corporation). Other calculations were performed in Excel (Microsoft Office 2016) and comparison of areas under ROC curves was made using MedCalc statistical software (Version 19.6.4, MedCalc Software Ltd, Belgium). Confidence level was defined at 95% for all statistical tests.

## Results

Out of the 58 teeth initially included for examination, 5 either did not fulfill the study’s inclusion criteria after tooth extraction and second inspection in vitro, or were destroyed while sectioning for histological analysis. Finally, a total number of 53 teeth with 118 examination sites were included for statistical analysis. Out of those, some examination sites could not be assessed using the algorithms, either due to insufficient scan data or algorithm failure; the number of missing examination sites for each algorithm can be seen in the contingency tables (Supplementary table S1). According to histology, out of the total number of examination sites ($n=118$), 17 were sound (E0), 79 were initial caries lesions in enamel (E1, E2), 8 were lesions in the outer third of dentin (D1) and 14 were lesions located in the middle-inner third of dentin ($\ge$ D2).

### Diagnostic performance

**Figure 2:**
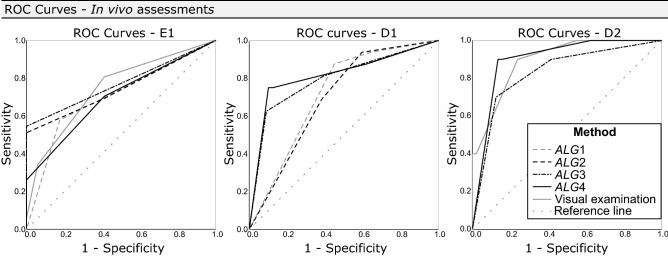
ROC curves corresponding to the four algorithms (*ALG1–ALG4*) and visual assessment investigated in vivo at the histological levels E1, D1, D2. On the ROC curves, the Sensitivity values are plotted against the 1-Specificity values. The curves closer to the top-left corner indicate a better diagnostic performance, while curves closer to the reference diagonal line indicate inferior diagnostic performance.

Table 2 shows descriptive results including correlation to the histological scores $(r_s)$, Az, SE, SP and ACC for all algorithms, both in vivo and in vitro, and for visual examination in vivo. Figure 2 presents the ROC curves corresponding to algorithms and visual examination in vivo. All methods resulted in significant correlation ($r_s$) with histology ($p<0.001$): *ALG3*, *ALG4* and visual assessment showed moderate correlation (0.41 $\le$ $r_S$ $\le$ 0.54) and *ALG1* and *ALG2* showed fair or weak correlation ($r_S<0.40$) Table 2.

**Table 2:** Descriptive results for all methods assessed in vivo (a) and in vitro (b). $r_s$, Spearman’s rank correlation coefficient; Az, area under the ROC curve; SE, sensitivity—true positive rate; SP, specificity—true negative rate; ACC, diagnostic accuracy; N/A, not available. Standard error is provided in parenthesis. SE and SP standard error is adjusted for clustered data^32^. The significant differences within the same row are marked with capital letters following the sequence $A>B>C$. Confidence level was defined at 95% for all statistical tests.

		(a) In vivo				
		*ALG1*	*ALG2*	*ALG3*	*ALG4*	Visual
	$r_s$	0.37 (0.08)	0.39 0.08)	0.50 0.08)	0.46 (0.08)	0.54 (0.07)
$\ge$E1	Az	$0.71 (0.06)^A$	$0.75 (0.05)^A$	$0.78 (0.05)^A$	$0.71 (0.06)^A$	$0.76 (0.06)^A$
	SE	$0.74 (0.05)^{AB}$	$0.70 (0.05)^B$	$0.56 (0.06)^C$	$0.71 (0.05)^{AB}$	$0.82 (0.03)^A$
	SP	$0.53 (0.03)^B$	$0.59 (0.02)^B$	$1.00 (0.00)^A$	$0.59 (0.03)^B$	$0.59 (0.04)^B$
	ACC	0.70	0.68	0.63	0.69	0.79
$\ge$D1	Az	$0.73 (0.06)^A$	$0.70 (0.06)^A$	$0.81 (0.06)^A$	$0.83 (0.06)^A$	N/A
	SE	$0.89 (0.10)^A$	$0.71 (0.16)^A$	$0.67 (0.12)^A$	$0.78 (0.13)^A$	N/A
	SP	$0.55 (0.03)^C$	$0.62 (0.02)^B$	$0.91 (0.02)^A$	$0.88 (0.02)^A$	N/A
	ACC	0.61	0.63	0.87	0.86	N/A
$\ge$D2	Az	N/A	N/A	$0.81 (0.08)^A$	$0.90 (0.04)^A$	$0.90 (0.04)^A$
	SE	N/A	N/A	$0.73 (0.15)^A$	$0.91 (0.19)^A$	$0.93 (0.07)^A$
	SP	N/A	N/A	$0.88 (0.02)^A$	$0.86 (0.02)^A$	$0.75 (0.02)^B$
	ACC	N/A	N/A	0.86	0.87	0.77

		(b) In vitro				
		*ALG1*	*ALG2*	*ALG3*	*ALG4*	
	$r_s$	0.37 (0.08)	0.20 (0.09)	0.41 (0.08)	0.47 (0.08)	
$\ge$E1	Az	$0.70 (0.08)^A$	$0.70 (0.08)^A$	$0.70 (0.06)^A$	$0.76 (0.05)^A$	
	SE	$0.85 (0.03)^A$	$0.81 (0.03)^A$	$0.59 (0.05)^B$	$0.80 (0.04)^A$	
	SP	$0.53 (0.03)^A$	$0.59 (0.03)^A$	$0.71 (0.02)^A$	$0.59 (0.03)^A$	
	ACC	0.80	0.77	0.60	0.77	
$\ge$D1	Az	$0.73 (0.05)^A$	$0.58 (0.07)^B$	$0.81 (0.06)^A$	$0.83 (0.05)^A$	
	SE	$1.00 (0.00)^A$	$0.47 (0.16)^B$	$0.72 (0.13)^{AB}$	$0.74 (0.12)^{AB}$	
	SP	$0.46 (0.02)^C$	$0.58 (0.02)^B$	$0.86 (0.02)^A$	$0.81 (0.02)^A$	
	ACC	0.55	0.56	0.84	0.72	
$\ge$D2	Az	N/A	N/A	$0.85 (0.05)^A$	$0.85 (0.05)^A$	
	SE	N/A	N/A	$0.82 (0.14)^A$	$0.83 (0.12)^A$	
	SP	N/A	N/A	$0.83 (0.02)^A$	$0.83 (0.02)^A$	
	ACC	N/A	N/A	0.83	0.83	

#### Caries detection level (Histology $\ge \mathbf {E1}$)

When assessing the ability of the different investigated methods to detect caries lesions in vivo and in vitro ($$\mathrm {Histology\ge E1}$$), all methods resulted in similar area under the ROC curves (Az); no significant differences among the Az values of different methods were observed ($$p>0.05$$). The highest SE and ACC were exhibited by *ALG1*, *ALG4*, and visual assessment, while significantly lower SE was found for *ALG3* ($$p<0.001)$$. However, *ALG3* presented the highest SP ($$p<0.05$$) in vivo. No significant differences among SP values were observed in vitro.

#### Caries in dentin (Histology $\ge \mathbf {D1}$)

As regards the detection and classification of caries lesions in the outer third of dentin ($\mathrm {Histology\ge D1}$), both in vitro and in vivo, only the IOS algorithms were assessed as there is no ICDAS score for visual examination that can reliably distinguish between lesions in enamel and initial lesions in the outer third of dentin^24^. When assessing the 3D models acquired in vivo, no significant difference among the Az for all algorithms was detected ($p>0.05$). However, regarding measurements on models obtained in vitro, *ALG2* resulted in significantly lower Az values than in vivo ($p<0.01$). *ALG3* and *ALG4* showed the highest Az, SP, and ACC both in vitro and in vivo. On the other hand, *ALG*1 exhibited significantly lower SP ($p<0.05$) than all the other algorithms, but high SE.

#### Caries in the middle-inner third of dentin (Histology $\ge \mathbf {D2}$)

In the middle-inner third of dentin ($\mathrm {Histology\ge D2}$), only the in vivo visual scores, and those from *ALG3* and *ALG4* were assessed. Regarding the Az and SE values, no significant differences among the different methods were observed. Visual assessment showed the lowest ACC and SP in vivo, with the latter being significantly inferior to the SP of *ALG3* and *ALG4*. Almost identical in vitro diagnostic performance was observed for *ALG3* and *ALG4*.

### Algorithm reproducibility in vivo vs. in vitro

No significant difference was found between in vivo and in vitro ordinal scores resulting from the IOS algorithms (McNemar Bowker test, $p >0.05$). In addition, for all algorithms and at all assessed histological levels (E1, D1, D2), no significant difference was detected between the Az values obtained from in vivo or in vitro assessments ($p > 0.05$).

However, as regards the caries detection level (Histology $\ge E1$), *ALG1*, *ALG2*, and *ALG4* showed higher SE in vitro than in vivo (McNemar’s test on binary data: *ALG1,2* $p<0.01$, *ALG4* $p=0.04$).

## Discussion

The algorithms for automated caries detection and classification defined for the IOS system (TRIOS 4, 3Shape TRIOS A/S, Denmark) were validated against histology. This study is a significant step towards implementing an automated caries scoring system in a commercial 3D IOS system, which can aid caries detection and potentially support caries monitoring in everyday clinical practice. When considering the detection and classification of initial ($\mathrm {Histology\ge E1}$) and moderate-extensive caries lesions ($\mathrm {Histology\ge D2}$), the IOS algorithms showed diagnostic performance comparable to visual examination using ICDAS criteria; these results are in agreement with the literature assessing the QLF method^14,21^. The overall diagnostic performance of the different algorithms, as indicated by the area under the ROC curve, was similar for all the ALG in vivo and the visual assessment ($p > 0.05$). However, some statistical differences were observed among the SE and SP values at the different diagnostic levels ($p<0.05$). ALG1 and ALG4 showed similar SE and SP with the visual assessment, while ALG2 and ALG3 deviated, showing inferior SE at initial enamel caries lesions. Additionally, the ALG3 and ALG4 showed significantly higher SP regarding the more extensive dentin caries lesions (D2), where the visual assessment resulted in an increase number of false positive indications and inferior SP.

However, as expected in the current study and as also seen in the literature during validation of cut-offs defined for other devices^19–21^, i.e. applying cut-offs defined in a previous study on a new sample, the diagnostic performance of the investigated algorithms was considerably inferior to the one observed at optimal cut-offs assessed in a previous in vitro study^1^. This agrees with other studies supporting that no absolute cut-offs can be defined for the devices featuring optical caries detection with fluorescence. The defined cut-offs can only be used as an indication for the relative caries lesion depth^19–22^.

No significant overall difference was detected regarding the performance of the algorithms on the 3D models obtained in vivo or in vitro. This finding confirms that future caries validation studies assessing this IOS system can be conducted in vitro and provide a good indication of the in vivo diagnostic performance. Subsequently, caries classification cut-offs defined in vitro can potentially be applied in vivo. However, a prerequisite is that appropriate methodological procedures are followed in vitro after the tooth extraction, e.g. short storage period in liquid or freezing of teeth to avoid the diffusion of porphyrins in the storage solution^33^, fluorescence image acquisition in a dark room to avoid the effect of external light^27^.

Considering the level of subjectivity involved in the visual examination, and the documented influence of the individual examiner’s professional experience on its outcome^34^, the inclusion of only one examiner in this study might have introduced a level of bias. Significant discrepancies in this study’s results were likely to have been observed if more examiners, either with a different experience in Cariology research or general practitioners, had also conducted the visual examination^34^. Since the diagnostic performance of the visual assessment employing the ICDAS criteria is widely investigated in the literature, and the examiner variability is well known, this aspect was not addressed in the present study. In fact, a substantial level of reproducibility and accuracy is reported when calibrated, well-trained examiners employ the ICDAS criteria, such as in the current study^13,35^, and therefore, based on consensus, one trained and calibrated examiner can reliably conduct the visual examination alone^35^.

Some limitations are identified regarding the sample in this study. The fact that the investigated teeth were scheduled for extraction means that the majority were third molars, in some cases semi-erupted, or with large cavities. In addition, a few teeth were extracted for orthodontic reasons, or due to periodontal problems. The automated caries detection system is mainly intended to be used on permanent posterior teeth with initial to moderate caries lesions, for which monitoring can evaluate the progression or regression of the lesions, as well as the effectiveness of preventive measures. Thus, the constitution of the sample in the current study was not fully representative of the teeth in a clinical scenario. Nevertheless, this is an inherent limitation that, due to ethical considerations, could not be avoided in a validation study like this, where extraction and in vitro histological assessment were carried out.

Furthermore, the inclusion of third molars and semi-impacted teeth of limited clinical access created additional limitations, for example insufficient cleaning of the occlusal surface in some cases. Considering that the dental biofilm can emit strong red-orange fluorescence signal^36,37^, good cleaning of teeth is essential to assess caries lesions with the fluorescence method. Otherwise, the fluorescence signal from bacteria might lead to false indications by devices assessing fluorescence. This phenomenon became apparent in the current study as differences were observed in red fluorescence signal when assessing the scans of the same teeth obtained in vitro and in vivo (Fig. 1iib versus iiib). This variation in fluorescence signal resulted in higher SE values in vitro for the E1 histological level (*ALG1, ALG2, ALG4*). Additionally, in some cases, due to limited access of the scanner to third molars in vivo, areas of insufficient 3D scanning data (Fig. 1vi) on the occlusal surfaces were noted, thus leading to failure of the automated caries scoring algorithms. This is expected to be observed in a clinical setup as well, and the operators should be aware of such limitations.

Despite the good performance of IOS algorithms for caries detection and classification, there is still possibility for future algorithm improvement and implementation of other parameters, such as the surface area of caries lesions, in order to improve diagnostic performance. Incorporating the lesion surface area in the algorithms can potentially prevent the false classification of some narrow initial arrested caries lesions as more extensive lesions due to dark stains. There may also be potential in the assessment of caries lesion activity using this system by examining red fluorescence from the dental plaque, estimating lesion size change over time (i.e. monitoring), and obtaining information on surface roughness^38^, all worth investigating^39^. Lastly, the development of advanced algorithms based on machine learning seems promising given the recent advances in this field^9,10,12^.

## Conclusion

The automated algorithms for occlusal caries detection and classification accompanying the IOS system were validated against histology, showing an overall comparable in vivo diagnostic performance to the visual examination. The algorithms can be used both for in vitro and in vivo assessments. Only minor differences between their in vitro and in vivo diagnostic performance were noted.

This novel system exhibits encouraging performance for clinical application on occlusal caries detection and classification, while different approaches can be investigated for potential optimization of the system.

## Supplementary Information

Supplementary Information 1.

Supplementary Information 2.
