# Publisher Correction: Automated caries detection in vivo using a 3D intraoral scanner

**DOI:** 10.1038/s41598-021-01926-8

**Published:** 2021-12-08

**Authors:** Stavroula Michou, Mathias S. Lambach, Panagiotis Ntovas, Ana R. Benetti, Azam Bakhshandeh, Christos Rahiotis, Kim R. Ekstrand, Christoph Vannahme

**Affiliations:** 1 Department of Odontology, University of Copenhagen, 2200 Copenhagen, Denmark; 2 3Shape TRIOS A/S, 1060 Copenhagen, Denmark; 3 School of Dentistry, National and Kapodistrian University of Athens, 11527 Athens, Greece

Correction to: *Scientific Reports* 10.1038/s41598-021-00259-w, published online 28 October 2021

The original version of this Article contained an error in the layout of Table 1, where cells in the *ALG1,2* and Visual (ICDAS) columns were not merged correctly. The original Table 1 and accompanying legend appear below.

**Table 1:** Scoring systems employed by the different methods according to histology.

	Histology	*ALG1,2*	*ALG3,4*	Visual (ICDAS)
**SOUND**	**E0:** Sound	**0:** Sound	**0:** Sound	**0:** Sound tooth surfaces show no visible evi-dence of caries when viewed after cleaning and after 5 seconds of air-drying
**ENAMEL**	**E1:** Caries in the outer half of enamel	**1:** Caries in enamel	**1:** Caries in enamel	**1:** First visual change in enamel (opacity or discoloration) visible at the entrance of pit or fissure, seen after 5 seconds of air-drying
	**E2:** Caries in the inner half of enamel—including the dentin–enamel junction (DEJ)			**2:** Distinct visual change in enamel (opacity or discoloration) visible when both wet and dry, with no evidence of surface breakdown or underlying dentin shadowing
**DENTIN**	**D1:** Caries in the outer third of dentin		**2:** Caries in the outer third of dentin	
	**D2:** Caries in the middle third of dentin	**2:** Caries in dentin	**3:** Caries in the middle or inner third of dentin	**3:** A white or brown spot lesion with localized enamel breakdown, without visible dentin exposure
				**4:** Non-cavitated surface with an underlying dentin shadow, which obviously originated on the surface being evaluated
	**D3:** Caries in the inner third of dentin			**5:** Visually distinct cavity in opaque or discoloured enamel and exposed dentin
				**6:** Extensive (more than half of the surface) and visually distinct cavity with exposed dentin

The original Article has been corrected.

