# Author Correction: Automated caries detection in vivo using a 3D intraoral scanner

**DOI:** 10.1038/s41598-022-17576-3

**Published:** 2022-08-02

**Authors:** Stavroula Michou, Mathias S. Lambach, Panagiotis Ntovas, Ana R. Benetti, Azam Bakhshandeh, Christos Rahiotis, Kim R. Ekstrand, Christoph Vannahme

**Affiliations:** 1 Department of Odontology, University of Copenhagen, 2200 Copenhagen, Denmark; 2 3Shape TRIOS A/S, 1060 Copenhagen, Denmark; 3 School of Dentistry, National and Kapodistrian University of Athens, 11527 Athens, Greece

Correction to: *Scientific Reports* 10.1038/s41598-021-00259-w, published online 28 October 2021

The original version of this Article contained errors in the following sentence in the Results section.

“According to histology, out of the total number of examination sites (n=118), 28 were sound (E0), 51 were initial caries lesions in enamel (E1, E2), 31 were lesions in the outer third of dentin (D1) and only 8 were lesions located in the middle-inner third of dentin (≥ D2).”

now reads:

“According to histology, out of the total number of examination sites (n=118), 17 were sound (E0), 79 were initial caries lesions in enamel (E1, E2), 8 were lesions in the outer third of dentin (D1) and 14 were lesions located in the middle-inner third of dentin (≥ D2).”

The original Article has been corrected.

