# Supplementary material for: Automated caries detection in vivo using a 3D intraoral scanner
Source: Sci Rep. 2021 Oct 28;11:21276. doi: 10.1038/s41598-021-00259-w (PMC8553860; doi:10.1038/s41598-021-00259-w)
Supplement: Supplementary file 1 — Supplementary Information 1. [file 41598_2021_259_MOESM1_ESM.docx]

# Supplementary information

# “Automated caries detection in vivo using a 3D intraoral scanner.”

Contents

[Supplementary information: “Automated caries detection in vivo using a 3D intraoral scanner.” 1](#_Toc85375769)

[Supplementary methods 1](#_Toc85375770)

[Researchers’ blinding 1](#_Toc85375771)

[Researchers’ calibration 1](#_Toc85375772)

[Supplementary results 2](#_Toc85375773)

[Supplementary table S1. 2](#_Toc85375774)

## Supplementary methods

### Researchers’ blinding

The clinical examiner (P.N.) selected the examination sites, conducted the visual examination using ICDAS criteria and operated the 3D intraoral scanner to obtain the 3D models of the teeth, both *in vitro* and *in vivo.* This examiner was blinded to the results from the automated caries scoring system algorithms and histology.

The second examiner (S.M.) extracted the measurements from the 3D models using the four algorithms and conducted the histological analysis. The histological analysis was conducted before the assessment with the different algorithms. At that time, this examiner did not have access to the results from the visual assessment or the four algorithms. The same examiner scored the examination sites with the four algorithms at least two weeks after the histological analysis. Although blinding when extracting the scores for the four algorithms was not possible, we judge that this did not add any potential bias in the results, as the software automatically generated the scores without the examiner having any influence on it.

### Researchers’ calibration

The two researchers that conducted the *in vivo* and *in vitro* examinations (P.N., S.M.) were trained and calibrated prior to this study for each respective test.

When this study was conducted, the clinical examiner (P.N.) was a postgraduate student in restorative dentistry with at least five years of experience and clinical application of the ICDAS system. The examiner was first trained on an ICDAS educational software, and afterwards, he was calibrated by a certified ICDAS trainer on an independent sample of teeth. For the calibration purposes, the trainer and P.N. first scored ten teeth independently and then discussed the scores presenting disagreement until reaching an agreement. Afterwards, the trainer and P.N. scored a sample of 20 new teeth independently, and the agreement was found almost perfect (inter-examiner reliability, *κ* = 0.9). Finally, two weeks after the latter examination, P.N. re-assessed the sample of 20 teeth resulting in an intra-examiner agreement of *κ* = 0.85.

The second examiner (S.M.), at the time of the study, was a PhD student with four years of research experience within Cariology. S.M. was trained and calibrated to conduct the histological analysis at a previous study ^1^.

Furthermore, in the study mentioned above, the second examiner was trained to analyze 3D models and extract the caries classification scores from different algorithms (intra-examiner agreement, weighted kappa ≥ 0.9).

## Supplementary results

## Supplementary table S1.

Contingency tables presenting scores from the different algorithms *(ALG1-ALG4*) plotted against histological scores. Algorithm measurements obtained on *in vivo* and *in vitro* models and visual examination scores are presented*.* The scoring systems used are shown in the main article, **table 2**. The colored lines (red, green, yellow) represent the cut-offs corresponding to E1, D1 and D2 histological levels respectively.

Bibliography

1. Michou, S. *et al.* Development of a Fluorescence-Based Caries Scoring System for an Intraoral Scanner: An in vitro Study. *Caries Res.* **54**, 324–335 (2020).
