# Supplementary material for: Automated caries detection in vivo using a 3D intraoral scanner
Source: Sci Rep. 2021 Oct 28;11:21276. doi: 10.1038/s41598-021-00259-w (PMC8553860; doi:10.1038/s41598-021-00259-w)
Supplement: Supplementary file 2 — Supplementary Information 2. [file 41598_2021_259_MOESM2_ESM.pdf]

IOS algorithms - *In vivo*

Histology	Score	E0	E1	E2	D1	D2	D3	Total
ALG1 score	0	9	9	14	1			33
	1	5	1	11	1			18
	2	3	12	25	5	7	5	57
	Total	17	22	50	7	7	5	108
ALG2 score	0	10	9	17	1			37
	1	7	4	8	1	3		23
	2		9	25	5	4	3	46
	Total	17	22	50	7	7	3	106
ALG3 score	0	17	12	25	2		1	57
	1		8	19	1	2		30
	2							0
	3		2	6	4	5	3	20
	Total	17	22	50	7	7	4	107
ALG4 score	0	10	9	15	2			36
	1	7	10	27	1	1		46
	2			2				2
	3		3	6	4	5	5	23
	Total	17	22	50	7	6	5	107

IOS algorithms - *In vitro*

Histology	Score	E0	E1	E2	D1	D2	D3	Total
ALG1 score	0	9	4	10				23
	1	2	7	11				20
	2	6	13	31	7	7	5	69
	Total	17	24	52	7	7	5	112
ALG2 score	0	10	5	12			1	28
	1	3	8	16	3	3	2	35
	2	4	11	24	4	4		47
	Total	6	24	52	7	7	3	110
ALG3 score	0	12	13	24	2			51
	1	5	6	20	1	2		34
	2							0
	3		5	8	4	5	4	26
	Total	17	24	52	7	7	4	111
ALG4 score	0	10	6	13				29
	1	7	13	26	3	2		51
	2			5				5
	3		5	8	4	5	5	27
	Total	17	24	52	7	7	5	112

Visual

Histology	Score	E0	E1	E2	D1	D2	D3	Total
ICDAS score	0	10	7	11				28
	1	2	6	10				18
	2	4	6	20	2	1		33
	3	1	6	12	5	7		31
	4			1	1			2
	5					1	4	5
	6						1	1
	Total	17	25	54	8	9	5	118
